# Socio-demographic, behavioural and clinical factors influencing control of diabetes and hypertension in urban Mysore, South India: a mixed-method study conducted in 2018

**DOI:** 10.1186/s13690-022-00996-y

**Published:** 2022-11-15

**Authors:** Sudeshna Dey, Aparna Mukherjee, Manoj Kumar Pati, Arin Kar, Satyanarayana Ramanaik, Ashwini Pujar, Vidyacharan Malve, H. L. Mohan, Krishnamurthy Jayanna, Swaroop N

**Affiliations:** 1 Karnataka Health Promotion Trust (KHPT), Bengaluru, Karnataka 560044 India; 2 M. S. Ramaiah University of Applied Sciences, Bangalore, Karnataka 560054 India

**Keywords:** Diabetes, Hypertension, Associated multi-factors, Primary health care, Karnataka

## Abstract

**Background:**

Inadequate control of diabetes and hypertension is a major concern in India because of rising mortality and morbidity. Few studies in India have explored factors that influence control of diabetes and hypertension. The current study aimed to improve the understanding of multifactorial influence on the control of diabetes and hypertension among patients in Primary Health Care Settings(PHC) of urban Karnataka.

**Methods:**

We used a mixed-method study design, within a project aiming to improve non-communicable disease (NCD) continuum of care across PHC in Mysore city, India, conducted in 2018. The quantitative study was conducted among 399 patients with diabetes and/or hypertension and a logistic regression model was used to assess the factors responsible for biological control levels of diabetes and hypertension measured through Glycated Haemoglobin(HbA1c) and blood pressure. Further, in-depth interviews(IDI) were conducted among these patients and the counsellors at PHCs to understand the barriers and enablers for better control.

**Result:**

The quantitative assessment found odds of poor control amongst diabetics’ increased with older age, longer duration of disease, additional chronic conditions, and tobacco consumption. For hypertensives, odds of poor control increased with higher body mass index(BMI), alcohol consumption, and belongingness to lower social groups. These findings were elaborated through qualitative assessment which found that the control status was affected by stress as a result of family or financial worries. Stress, poor lifestyle, and poor health-seeking behaviour interplay with other factors like diet and exercise leading to poor control of diabetes and hypertension.

**Conclusion:**

A better understanding of determinants associated with disease control can assist in designing focused patient outreach plans, customized communication strategies, need-based care delivery plans, and specific competency-based capacity-building models for health care workers. Patient-centric care focusing on biological, social and behavioural determinants is pivotal for appropriate management of NCDs at community level in low-middle income countries.

## Background

India is experiencing a rapid demographic and epidemiological transition with Non-Communicable Diseases (NCDs), accounting for two out of every three deaths [1] and is the leading cause of preventable deaths in India. NCDs have reached an unprecedented level in India with diabetes and hypertension being the two major conditions affecting millions. In 2017, 74 million people in India were diabetic, which is projected to further increase to a staggering 134 million by 2045 [2]. Similarly, the number of hypertension cases in India is expected to increase from 118 million in 2000 to 214 million in 2025 [3]. These startling numbers are compounded by the fact that Indians suffer from diabetes and high blood pressure 5–10 years earlier than their western counterparts, leading to considerable economic losses to individuals and the nation as a whole [4].

Commitments to reduce the escalating burden of NCDs and their optimal management have attracted considerable attention in India. The Ministry of Health and Family Welfare (MoHFW), Government of India (GOI) launched the National Programme for Prevention and Control of Cancer, Diabetes, Cardiovascular Diseases and Stroke (NDCDCS) in July 2010 for prevention and control of NCDs. Strengthening of infrastructure including trained human resources, early diagnosis, management and integration with primary health care system through NCD cells to provide optimal operational synergies (NPCDCS, 2013) are some of the goals of this program. Despite these efforts at the national and state levels, implementation on the ground is affected due to the challenges at the facility, community and health system levels [5, 6].

In addition, the complex and multifactorial causation of NCDs further complicates the situation as observed in suboptimal control among diabetes and hypertension patients [7]. According to the World Health Organization (WHO), the fundamental way to control diabetes and hypertension is to address modifiable risk factors such as smoking, alcohol use, absence of physical activity, overweight and unhealthy diets [8]. These risk factors are further closely interlinked with socio-economic and structural determinants such as gender, age, wealth status, education, access to health care etc., often resulting in acute exacerbation, disabilities and the presence of co-morbidity [7, 9].

In India, a comprehensive program is needed to build an integrated system of disease prevention, health promotion, treatment, and care [10]. This can be possible through multisectoral actions and an understanding of “best buys” on population-based intervention. Therefore, it is important to design, implement, and evaluate the appropriate intervention in the care continuum for prevention and management of NCDs in primary health care settings [11–14]. Strengthening primary health care in urban areas through integrated service delivery can help in confronting the growing burden of NCDs [6, 15].

In this context, a collaborative initiative was undertaken by Karnataka Health Promotion Trust (KHPT) and Landmark Cares (the Social Initiative arm of Landmark Group in India) in consultation with the Department of Health and Family Welfare, Government of Karnataka, aimed at implementing a comprehensive model of NCD care-continuum at primary health care level in an urban area of Karnataka [15]. As part of this initiative, the intervention focused on two common NCDs- hypertension and diabetes. The intervention aimed at providing comprehensive information on lifestyle modification (diet, physical exercise, tobacco, alcohol) along with enhanced counselling on regular testing, physician consultations to diabetic and hypertensive patients, and strengthening referral facility at the Urban Primary Health Care Centre (UPHC).

GOI prepared long-term National Action Plan and Monitoring Framework that indicate out of ten targets to prevent and control NCDs, seven aim at reducing exposure to key risk factors [16]. Therefore, it is essential to assess the factors and barriers that influence diabetes and hypertension control both in the community and facility context [17–19]. This assessment is crucial for devising population-specific interventions and assimilation of knowledge on complex multifactorial associations with the disease [20, 21].

This study is aimed to understand factors and barriers to diabetes and hypertension control in UPHC in Mysore, South India. The study intended to add to the existing knowledge that can be used by the program managers to improve the quality of care at the facility and in community settings. The specific objectives of this study are, first, to understand the influence of socio-demographic, behavioural and clinical factors on the control of diabetes and hypertension; and, second, to learn about the perception, experience, barriers, and enablers to manage these chronic diseases.

## Methodology

### Study settings

This study was conducted in the Kumbarakoppalu UPHC area in Mysore City located around 90 miles away from the capital city of Bangalore in the southern state of Karnataka. The study was conducted among the population aged 30 years and above from June 2018 to September 2018. In 2017–18, during the first phase of the project in the same UPHC administrative area, a population-wide screening of diabetes and hypertension amongst those aged 18 years and above was conducted to assess the prevalence of diabetes and hypertension. The screening process covered 93% of the total population i.e. 32,596 aged 18 years and above residing in the UPHC area. Among them, 32,355 people were screened for diabetes and 32,568 people were screened for hypertension. The non-response rate for the screening of diabetes and hypertension together accounts for less than one percent i.e. 3200 people refused to participate in the screening process. We found the prevalence of diabetes and hypertension to be 12.12% and 19.43% among adults. The adults with diabetes comprise two categories. First, those who self-reported diabetes. Second, those who did not self-report but their Random Blood Sugar (RBS) level was found to be more than 140 mg/dl during the RBS test. Similarly, the adults with hypertension include both self-reported and screened populations. The screening follows the national guidelines which identify any adult with systolic blood pressure (SBP) above 140 mm Hg or diastolic blood pressure (DBP) above 90 mm Hg as hypertensive [15].

### Study methods and sample size

We used a mixed-method study design and weighted each method i.e. quantitative and qualitative based on the sequence of data collection and analysis [22]. The assessment was done to understand the factors affecting the biological control of diabetes and hypertension in terms of Glycated Haemoglobin (HbA1c) and Blood Pressure (SBP/DBP) levels respectively. Qualitative in-depth interviews (IDIs) with the patients and counsellors of the UPHC were conducted to understand the barriers and enablers for better control of hypertension and diabetes.

Cross-sectional design was adopted for quantitative study and to calculate sample size, results from the screening conducted in the first phase of intervention showed that 53% of the known diabetics (*N* = 2032) had RBS levels below 200 mg/dl, and 30% of the known hypertensive (*N* = 2122) had blood pressure levels under control (SBP < 140 & DBP < 90) [15]. Keeping these cut-offs, we hypothesised that the intervention would bring about an improvement in disease control by 15% point among diabetics and hypertensive by 2023. Considering a confidence interval (CI) of 95% with 80% power, the desired sample size was calculated as 207 and 202 people, for diabetes and hypertension respectively. Further, this was adjusted for non-response by 25% and the final sample size was determined as 250 each for diabetes and hypertension. The sampling frame of diabetics and hypertensive was stratified by gender and age before the individuals were selected systematically. The survey response rate was 88% and 99% for persons with diabetes mellitus and hypertension respectively.

For qualitative research, a purposive sample was drawn from the list of respondents who had participated in the survey. To understand how disease management and related behaviour varies, patients with controlled and uncontrolled levels of diabetes and hypertension were considered. A total of 16 patients with controlled and uncontrolled levels of diabetes and hypertension, with shorter (less than 5 years) and longer duration (more than 5 years) of these diseases and with or without other co-morbidities were interviewed. Recruitments ended after thematic saturation was obtained. Further, two community facilitators and one counsellor recruited by the project were also interviewed to understand their perspectives on key challenges that patients experienced in disease management. Community facilitators in the intervention program are involved both in mobilizing patients for screening at the UPHC and creating awareness related to disease management for the patients. The counsellor is based at the UPHC, whose main role is to provide counselling to the patients on diet, physical exercise and treatment regimen required to control hypertension/diabetes along with partaking the advanced tests (such as HbA1c, lipid profile, Albumin to Creatinine Ratio Test (ACR), retinopathy test, SBP and DBP and Electrocardiogram (ECG)).

### Data collection and measurements

The quantitative data was collected using a structured interview schedule. All interviews were conducted in the local language by trained field researchers. The study tool was initially developed in the English language, translated to the local language Kannada and translated back to English to ensure the validity of the tool. The Kannada study tool was piloted and revised before the actual survey. The study tool was a local adaptation of the who's STEPS NCD questionnaire [23]. The study tool was developed to capture crucial patient information such as medication history and treatment adherence in addition to demographic, social and economic characteristics; vital information on tobacco use, alcohol consumption, diet pattern, and level of physical activity was also collected. Information on caste was collected and considered as a proxy of socio-economic status identified as Schedule Caste (SC), Schedule Tribe (ST), Other Backward Classes (OBC) and others. Post administration of the questionnaire, the respondents were mobilised to the UPHC to get the HbA1c and blood pressure test done at their preferred time. Those who did not turn up for the test even after participation in the survey were excluded from the final analyses. Anthropometric measurements of weight and height were measured with a digital weighing machine (Omron HN-286-AP) and stadiometer (Prestige) respectively by a trained nurse at the facility. While the HbA1C test was done through a compact rapid multi-assay analyser (Alere Afinion) at the UPHC, blood pressure was measured using a digital sphygmomanometer (Omron HEM 7120). Three blood pressure readings were taken and the higher of the last two values were considered for analysis.

Information on tobacco consumption was collected for both smoking and smokeless variants. Those who consumed tobacco regularly in the past 30 days were considered as ‘current’ users. Similarly, current alcohol consumption was defined as self-reported use of alcohol in the past 30 days. Serving sizes of fruits and vegetables in a typical week were determined using show-cards and measuring bowls. To measure physical activity, time spent on moderate and/or vigorous physical activity during work, leisure time and commutation was converted into minutes per week and later converted to Metabolic Equivalent Task (MET)-minutes per week (one minute in moderate and transport-related activities equal to 4 MET-minutes and one minute in vigorous activities equal to 8 MET-minutes) [24].

Subsequently, qualitative research was undertaken in September 2019 using a semi-structured in-depth interview guide. Along with this a detailed history of medical treatment was also captured by taking pictures of all the available medical records and prescriptions for each of the respondents.

Interviews consisted of open-ended questions that aimed to elicit participant experiences with their disease and its management. A semi-structured interview guide was used to facilitate the IDIs with topics encompassing perception related to health, hypertension, and diabetes-specific health issues broadly covering diagnosis, understanding of the disease, treatment and its management, family and social support, major life events, other co-morbidity, and overall wellbeing. For all the patients, complete socio-demographic information was also collected.

### Analysis

Descriptive statistical analysis was used to understand the socio-demographic characteristics (age, gender, caste, education, employment, wealth score), behavioural practices (fruit and vegetable intake, physical activity, consumption of alcohol, consumption of tobacco), and clinical characteristics (duration of disease, presence of co-morbidity, BMI) among diabetics and hypertensive by their biological disease control status. Multivariate logistic regression was used to assess factors affecting biological control among diabetics and hypertensive. The key outcome variables were status of control among diabetics and hypertensive (good control vs poor control). This categorisation is based on American Diabetes Association (ADA) guidelines for age-standardized HbA1c cut-offs where, HbA1c < 7 among adults < 65 years, and HbA1c < 7.5% among elderly 65 years and above are considered as good control and HbA1c > 7and > 7.5 as poor control [25]. Similarly, the control level for blood pressure among hypertensives follows the standard cut-offs provided by the Eighth Joint National Committee (JNC 8), where., SBP < 150 mm of Hg and DBP < 90 mm of Hg among people ≥ 60 years of age and SBP < 140 mm of Hg and DBP < 90 mm of Hg among people < 60 years of age are considered in good control and vise versa for poor control level [26].

The socio-demographic, behavioural and clinical risk factors were included as the predictor variables in the model. We developed Socio-Economic Status (SES) scores using house type and Standard of Living (SLI) characteristics as per the National Family Health Survey-4 (NFHS-4) [2] and generated wealth scores using the principal component analysis method. Further, the duration of the disease was obtained from information on the onset of the disease. Comorbidity metrics was constructed based on information on any chronic diseases like dyslipidemia, chronic kidney disease, tuberculosis, coronary artery disease, stroke, congestive heart failure, and diabetic retinopathy. The variable of comorbidity was defined as a person suffering from diabetes or hypertension along with at least one disease as mentioned earlier. The Body Mass Index (BMI) was calculated as weight in kilograms divided by height in meters squared [27]. The thresholds for BMI were defined as < 25 kg/m2 (Normal), 25 kg/m2 and above (Overweight/Obese). Since a very small number of respondents reported consuming five or more servings of fruits and vegetables in the sample as recommended by WHO for healthy dietary practice, we categorised servings consumed into three and more number of servings. For physical activity, MET-minutes were divided into inappropriate (< 600 MET-minutes/week) and appropriate (≥ 600 MET-minutes/week) physical activity categories. All the statistical analysis was performed using Stata software version 14.0.

A team of three qualitative researchers analysed the qualitative data. All interviews were audio-recorded and transcribed verbatim. All the transcribed verbatim in the local language (Kannada) was then translated to English. We conducted a thematic analysis and broadly followed the approach by Braun and Clarke [28]. First, the three qualitative researchers familiarized themselves with the data by thorough reading and re-reading processes. Each researcher independently read the transcripts, coded and analysed the qualitative data, and these were further discussed and checked for accuracy. Once completed, the initial codes were created by using an inductive-deductive approach. For example, codes included background characteristics, daily routine and lifestyle, disease prevention, treatment and management, alternative remedies, community perception of the disease, misconceptions around the disease and its treatment, challenges in disease management, co-morbidity, long-term impact of the disease on the health etc. Then by collating the codes the emerging themes were identified, such as stress, lifestyle related behaviour, inconsistencies in treatment, medication and follow-up, age-specific behaviour, and family support. The patterns within the theme were further reviewed and evaluated between and within the respondents with poor control and good control of the disease. Lastly, the researchers tried to organize and ensure the coherence of the identified themes concerning the research questions. Similar patterns that were notably emerging from patients with poor control were joined in a group under different themes and were compared to those under good control. The three independent researchers again discussed the emerging themes, findings and potential biases or subjectivity to ensure deep understanding and triangulation of the data. Further, relevant literature was reviewed and referred to strengthen our triangulation process. The final set of three major themes was identified, and accordingly, the qualitative findings are presented in the form of excerpts and narratives.

### Ethics statement

Ethics approval was obtained from the ethics board of GRAAM (Grassroots Research and Advocacy Movement) Mysore, approval number IRB (Health) No: 02/2017–18 and IRB (Health) No: 062019–20. All the study participants were contacted through the project team and were explained the study objectives. Written informed consent was obtained from those who agreed to take part in the study before the interviews. Consent for audio recording and capturing snapshots of medical records/prescriptions/images were also obtained for those who participated in the qualitative interviews. Anonymity was maintained by creating a unique study ID. As part of the consenting procedure, participants were assured that their participation was voluntary, and their decision to participate or decline will not affect their health care entitlements.

## Results

### Summary statistics

Baseline socio-demographic, behavioural and clinical characteristics of diabetics and hypertensive by their biological control status are presented in Table 1. Overall, 58.70% of hypertensive and 29.03% of diabetics achieved biological control levels. 79.3% of long-term diabetics (more than 10 years of history) were not under biological control, while 61.8% among recently diagnosed diabetics (less than five years) were under control. Similarly, diabetics with other co-morbidities had poor control levels than those without (85.3% vs. 68.3%). In contrast, among the hypertensive, no such associations are observed. Obesity remains one of the major risk factors; 72.2% of obese diabetics and 47.3% of obese hypertensive did not achieve good control. We found no association between alcohol consumption and blood sugar levels; however, 57.3% of hypertensive who consumed alcohol did not achieve good blood pressure control. Apart from clinical and behavioural factors, socio-economic factors like caste and work status showed some association with control level. While 70.7% SC/STs had their blood pressure under control, this value was 56.3% among other groups. No such caste association was seen among the diabetic. Conversely, diabetics with more years of education had better blood sugar control, but no such association was seen among hypertensive. In terms of employment status, 51.3% of hypertensive who were engaged in any paid job/remunerative work had their blood pressure under control as compared to 61.85% who were not employed. No such difference was evident among people with diabetes.

**Table 1:** Characteristics of study participants, disaggregated by control levels for diabetes and hypertension (Mysore city, Karnataka state, India), 2018

Variables	Diabetes		Hypertension	
	**Good control**	**Fair/poor control**	**Controlled**	**Uncontrolled**
	**Frequency (%)**	**Frequency (%)**	**Frequency (%)**	**Frequency (%)**
**HbA1c**	63 (29.03)	154 (70.97)	-	-
**SBP/DBP**	-	-	145 (58.7)	102 (41.3)
**Mean (Std. dev.) Age**	59.41 (11.38)	55.80 (10.74)	58.45 (11.93)	56.77 (10.44)
**Gender**				
Male	32 (30.77)	72 (69.23)	58 (58.59)	41 (41.41)
Female	31 (27.43)	82 (72.57)	87 (58.78)	61 (41.22)
**Caste**				
OBC/Others	55 (29.1)	134 (70.9)	116 (56.31)	90 (43.69)
SC/ST	8 (28.57)	20 (71.43)	29 (70.73)	12 (29.27)
**Education**				
No schooling	20 (28.99)	49 (71.01)	53 (62.35)	32 (37.65)
Till 10th standard	27 (25.23)	80 (74.77)	64 (55.65)	51 (44.35)
11th and above standard	16 (39.02)	25 (60.98)	28 (59.57)	19 (40.43)
**Employment**				
Unemployed	40 (30.08)	93 (69.92)	107 (61.85)	66 (38.15)
Employed	23 (27.38)	61 (72.62)	38 (51.35)	36 (48.65)
**Duration of disease**				
< 5 years	34 (38.2)	55 (61.8)	56 (58.33)	40 (41.67)
5 to < 10 years	12 (26.09)	34 (73.91)	34 (56.67)	26 (43.33)
10 years and above	17 (20.73)	65 (79.27)	55 (60.44)	36 (39.56)
**Presence of co-morbidity**				
No	58 (31.69)	125 (68.31)	122 (58.1)	88 (41.9)
Yes	5 (14.71)	29 (85.29)	23 (62.16)	14 (37.84)
**BMI status**				
Normal	21 (31.82)	45 (68.18)	48 (76.19)	15 (23.81)
Over weight/Obese	42 (27.81)	109 (72.19)	97 (52.72)	87 (47.28)
**Fruits and veggies intake**				
No fruits and vegetables	24 (38.1)	63 (72.41)	44 (52.38)	40 (47.62)
< 3 Servings	34 (33.01)	69 (66.99)	77 (60.16)	51 (39.84)
3 and more servings	5 (18.52)	22 (81.48)	24 (68.57)	11 (31.43)
**Physical Activity**				
Appropriate	29 (24.37)	90 (75.63)	86 (63.24)	50 (36.76)
Inappropriate	34 (34.69)	64 (65.31)	59 (53.15)	52 (46.85)
**Consumption of alcohol**				
No	51 (28.18)	130 (71.82)	134 (60.63)	87 (39.37)
Yes	12 (33.33)	24 (66.67)	11 (42.31)	15 (57.69)
**Consumption of tobacco**				
No	61 (31.44)	133 (68.56)	135 (58.44)	96 (41.56)
Yes	2 (8.7)	21 (91.3)	10 (62.5)	6 (37.5)

Note: Age-standardised cut-offs were considered for HbA1c and blood pressure levels; HbA1c < 7 among adults < 65 years, and HbA1c < 7.5% among elderly 65 years and above are considered as good control. SBP < 150 mm of Hg and DBP < 90 mm of Hg among people ≥ 60 years of age and SBP < 140 mm of Hg and DBP < 90 mm of Hg among people < 60 years of age are considered good control. Caste- OBC as Other Backwards Classes, Others as any other classes; SC as Schedule Caste and ST as Schedule tribe

### Regression results

Tables 2 present the effect of socio-demographic, behavioural and clinical indicators on the control of diabetes (HbA1c levels) and blood pressure (SBP/DBP levels) respectively. The final regression model consists of 217 diabetics and 247 hypertensives patients. The regression results revealed that with one unit increase in age, a diabetic patient was less likely to have poor control by a significant odds of 0.96 (CI: 0.926–0.996) than those of a younger diabetic. SC/ST group exhibits lower odds of uncontrolled SBP/DBP levels compared to other backward caste or general caste patients (0.48, CI: 0.222–1.059), however the estimates for confidence interval suggest that the SC/ST group can exhibit higher odds. Therefore, no conclusion can be drawn from the estimated odds ratio. Clinical factors including duration of onset of disease, presence of co-morbidity, and BMI status were estimated. Duration of disease was directly proportional to poor glycaemic control among diabetics; while longer duration such as more than 10 years duration (2.54, CI: 1.085–5.964) had significantly higher odds of being in poor control compared to recently diagnosed diabetics (duration less than 5 years). Although the odds for 5–10 years duration is significant at a 10% level (2.17, CI: 0.887–5.309), therefore no conclusion can be drawn for the same as the lower value of confidence interval suggests that the odds can be lower. Diabetics with other chronic conditions were about three times (CI: 1.145–8.311) more likely to have poorly controlled blood sugar compared to those without other chronic conditions. People consuming tobacco products whether smoke or smokeless had a higher likelihood (8.32, CI: 2.147–32.282) of having poor control similarly people categorised as overweight or obese were at higher risk (2.91, CI: 1.526–5.550) for uncontrolled SBP/DBP levels compared to those with normal weight. Although the estimated odds ratio for alcohol consumption (CI: 0.975–7.079) shows an increased risk for uncontrolled SBP/DBP levels, however, no conclusion can be drawn as the lower estimate of confidence interval suggest that the odds can be lower as well.

**Table 2:** Factors associated with poor control/uncontrolled status of diabetes and hypertension obtained from multivariate analysis (Mysore city, Karnataka state, India), 2018

**Variables**	**Diabetes**		**Hypertension**	
	**Odds-Ratio [95% CI]**	***P*-Value**	**Odds-Ratio [95% CI]**	***P*-Value**
**Age**	0.960 [0.926–0.996]	0.027	0.996 [0.967–1.026]	0.779
**Gender**				
Male				
Female	1.309 [0.513–3.344]	0.573	1.729 [0.791–3.779]	0.17
**Caste**				
OBC/Others				
SC/ST	1.329 [0.488–3.623]	0.578	0.484 [0.222–1.059]	0.069
**Employment**				
Unemployed				
Employed	1.229 [0.482–3.133]	0.666	1.631 [0.754–3.528]	0.214
**Education**				
No schooling				
Till 10th standard	1.121 [0.462–2.719]	0.801	1.120 [0.574–2.186]	0.74
11th and above standard	0.568 [0.204–1.585]	0.28	1.036 [0.423–2.538]	0.939
**Wealth Score**	1.248 [0.888–1.754]	0.203	0.978 [0.730–1.310]	0.883
**Duration of disease**				
< 5 years				
> = 5 to < 10 yr	2.171 [0.887–5.309]	0.089	1.219 [0.600–2.474]	0.584
> = 10 yr	2.544 [1.085–5.964]	0.032	0.969 [0.491–1.913]	0.929
**Presence of co-morbidity**				
No				
Yes	3.085 [1.145–8.311]	0.026	1.305 [0.744–2.289]	0.353
**BMI status**				
Normal				
Over weight/Obese	1.328 [0.657–2.684]	0.43	2.911 [1.526–5.550]	0.001
**Fruits and vegetable intake**				
No fruits and veggies				
< 3 servings	0.949 [0.463–1.945]	0.886	0.682 [0.370–1.257]	0.22
3 and more servings	2.078 [0.609–7.094]	0.243	0.566 [0.225–1.425]	0.227
**Physical Activity**				
Appropriate				
Inappropriate	0.719 [0.361–1.431]	0.347	1.526 [0.859–2.712]	0.149
**Current consumption of tobacco**				
No				
Yes	8.325 [2.147–32.282]	0.002	1.063 [0.359–3.145]	0.912
**Current consumption of alcohol**				
No				
Yes	0.749 [0.298–1.882]	0.539	2.628 [0.975–7.079]	0.056
Constant	9.128 [0.683–121.954]	0.095	0.215 [0.023–2.045]	0.181
**Observations**	217		247	
**Log pseudolikelihood**	-112.78911		-153.32914	

Statistical significance at the level of 10%, 5% and 1% respectively

### Results from qualitative study

Patients who participated in the qualitative interviews (n = 16) included 10 patients with poor control and 6 with good control levels of diabetes/hypertension. Of the total 16 participants, 8 were male and 8 were female. Three respondents were between the age group 40–50 years while six were between 51–60 years and the remaining were 60 and above years old. Nine respondents had both diabetes and hypertension while 4 had diabetes and 3 had hypertension only. Five patients were having their duration of illness between 5–10 years while others had more than 10 years. Only one patient reported having been diagnosed in the last one year. 10 patients have co-morbid chronic illness, of which six patients had heart-related complications while the remaining had health complications related to kidney and vision.

### Barriers to good biological control of diabetes and hypertension

#### Stress

The qualitative study findings pointed out that stress is one of the key barriers for controlled diabetes and hypertension and the narratives of all cases in the study suggested that stressful life events often affect diabetes/hypertension in its exacerbation.

We found that most of the cases were associated with hidden and persistent emotional burdens and worries related to family issues or financial problems. Generally, women were more stressed due to family problems and men due to financial worries. A review of the medical records also revealed that during stressful life events patients experienced a considerable spike in their blood pressure and blood sugar values. Experiences of stress might have led to poor self-care, poor health-seeking behaviour and opting for unhealthy behaviours, such as consumption of alcohol and smoking, which in turn pose a greater risk to develop diabetes and hypertension-related health complications. *R1* recalled how the stress or anxiety due to economic hardship, loneliness and physical injury led to difficulties with self-care, which manifested through an unhealthy diet, less physical activity, or difficulties with taking the medication regularly.

I used to have agricultural land but I sold it to get my daughter married. I have so much debt on me. …I stay away from my family. I still have 4 other family members who depended on me. I had an accident a few years back, since then my spinal cord hurts, …I always feel guilty as I am unable to provide a good life for my family. Here there is no one to talk to. To forget my worries, I just drink alcohol day and night, it is my only friend. I have had diabetes for 19 years, I don’t remember when I checked it last. I just take the insulin injections whenever I feel extremely tired (R1, male 53 years, diabetes, not under control)

The following narratives from our study *participants R2* and *R3* respectively provide an example of how women become emotionally drained and tend to resort to poor eating habits and irregular medication while men took up smoking and alcohol as a common way to deal with their stress.

“I have faced problems throughout my life, I lost one of my sons, my other son is handicapped, he doesn’t earn…. he and his family are completely dependent on us, and my younger son stays separately married to a girl who often quarrels with us and constantly threatens us for the property. My husband is also retired….So I have to manage within our savings….I have also undergone heart surgery…what else …I am taking medicines…with all these worries how do you expect me to do anything else?”- (R2, Female, 59 years, both diabetes and hypertension, not under control)

“I have both diabetes and hypertension, it was manageable, …I was working in a tire company but in 2008 I lost my job …my blood pressure and blood sugar values shot up…I had a huge debt and no job with two children to take care. My son never supports me, just roams around, daughter is studying in a college and yet to be married….I am 49 years…how can I find a new job…ultimately after struggling a lot I got a job as a security guard. I take medicines at times, but I don’t have any time to do exercise… I smoke too much to reduce the stress….my job schedule doesn’t allow me to have food on time and the doctor says my blood pressure, and blood sugar levels are still very high….”(R3, Male, 49 years, both diabetes and hypertension, not under control)

The above narratives clearly show that the behavioural mechanisms through which stressful experiences might affect diabetes or hypertension control are varied and often complex. Irrespective of better knowledge of the disease and awareness of one own disease status or medication, physiological reactions to external stressors may negatively affect diabetes or hypertension control. Though the mechanisms of poor outcomes of stress on diabetes are not very clear but it may lead to difficulties with self-care manifested through less physical activity, poorer diet, or difficulties with taking medication.

Even findings from the study show that most of the patients who came to the UPHC for care and treatment more often discussed their family problems with the counsellor along with health-related issues. Further, we found that many patients, such as elderly men and women, widowed, retired who were dependent on others faced challenges in disease management due to lack of family and social support. The counsellor emphasized the need to prioritize patients with poor control levels and conduct patient counselling along with the family for better disease management. In addition to clinical counselling, counselling needs with regards to mental health issues existed for most of the patients suggesting for service of dedicated mental health counsellor. Moreover, doctors gave very little time to the patients to explain their concerns regarding the disease and queries about its management.

*“ There is no provision of counsellor at the UPHC...I only do all the testing and provide lifestyle advice to the patients. Many patients have family problems or some other financial problems, they start crying in front of me… whatever advice I give them also needs involvement and constant support from their families but it is difficult to expect anything from them….”* (*R4, female counsellor from the intervention program*)

### Poor lifestyle

Poor lifestyle choices, such as a meager diet, smoking, overuse of alcohol, and lack of physical activity often fuel poor health and further deteriorate the health conditions like diabetes and hypertension. The risk with a poor lifestyle is that it gradually becomes a way of life, and may not look risky in short term but have far-reaching harmful effects in the long term. This study found that various factors were contributing to the poor lifestyle. The below narratives of *R5* and *R6* reflect the common barrier to a healthy diet and it has been a perceived lack of control over eating patterns and preferences which can further be linked to a lack of intrinsic motivation to regulate their eating behavior.

“I know I have sugar (diabetes), I should restrict intake of certain food items, if I wish to eat I will eat and I don't care about anything…. when my family members are not there I put some extra sugar into my coffee, otherwise, they give me low sugar coffee….I like sweets and I will have them” (R5, Male, 69 years, both diabetes and hypertension–not under control)

“Our mind will always be fickle and we can’t stop it completely. Whenever I want to have it (alcohol) I go with my friends and have it….what is wrong in that” (R6, male, 53 years, diabetic, not under control)

Many respondents felt that taking medicine was enough and one need not necessarily follow the diet or any physical activity regimen. Many pointed out that doing household work or traveling for work was sufficient to control their blood pressure or sugar levels.

I am doing so much work at home, I start working from 5 in the morning. You can imagine how much time I have to spend cleaning the home and cooking food for 6 people in the family. Where do I have the time to do anything else?? (R7, female, 41 years, diabetes and hypertension-uncontrolled)

People do so many things like …food restrictions, exercise, walking, gym, yoga and still they take medicine…so how does it make any difference… I feel as long as I am taking medicines on time, nothing else is needed. Household chores are themselves too much and there is no need for exercise. Better eat and enjoy everything and just take medicines along with it. (R7, female, 41 years, diabetes and hypertension-not under control)

I don’t do anything like that, we just need to do some walking …and that much I think gets covered when I walk to my workplace or go to market… (R8, male 57 years, diabetic, not under control)

Other factors like the sedentary nature of work, high and frequent consumption of non-vegetarian food, lack of time and space to go out for walking or exercise contributed to a poor lifestyle. Many of the women respondents shared that taking out time from their household duties and also lacking space to go out sometimes demotivated them to go out for a walk or to do exercise. In-depth analysis shows that due to lack of space women were hesitant and shy to go into public spaces to exercise. In addition, narratives also suggested that open and safe spaces in the city are also a decisive factor for patient choices to do physical exercise such as walking, running or exercise.

“We practice “gudde mamsa” here...as a result of almost every weekend we eat red meat and it’s a rich preparation...you tell me how can one resist meat when my family and neighbours are enjoying so much of non-veg.” (R9, male, 65 years, both diabetes and hypertension-not under control)

*“I work in a sweet shop and I make sweets all day long…for this, I have to sit in one place constantly…I don’t get the time to do any physical activity, I am under medication and eat according to the doctor’s advice, but still, my* blood pressure *is high…what to do, should I leave my job….” (R10, male, 39 years, diabetes, not under control)*

“In this neighborhood, we don’t have any parks or footpaths to walk. Walking on the streets between the houses is not comfortable, everywhere there are cars and bikes parked on the streets…moreover, people are driving so badly nowadays… Who will go to a park away from home, who has that much time… I am taking my medicines and I go to work, I think that is enough for me....”( R9, male, 65 years, both diabetes and hypertension-not under control)

### Poor health-seeking behaviour

Most of the respondents were taking medicines irregularly leading to poor biological control. Unfolding the reasons for poor adherence were rooted in perceived treatment efficacy, medication beliefs, trust in one’s health care providers, unavailability of medicines at the UPHC, and poor doctor-patient rapport. *R11’*s insulin-taking practices reflect the poor patient understanding of the disease and its management.

*“I keep the insulin in one glass of water because we don’t have a refrigerator at my home. whenever I go to work, … I just carry the insulin in my pocket. After having my food outside I will just inject the insulin myself and complete my work and return home* ***”*** *(R11, male, 57 years, diabetic, not under control)*

Since diabetes and hypertension are chronic diseases that require lifelong treatment and management, many patients shared that they often feel disappointed and annoyed about maintaining a strict regimen leading to dietary negligence and improper medication. Also, because of limited knowledge and understanding of the disease and medications, few respondents reported taking medicine only when they felt the need.

“I come to know of my higher biological levels whenever I feel tired and then I take one tablet extra and I will feel better” (R5, Male, 69 years, both diabetes and hypertension, not under control)

“Help me understand what’s going on with me…what do the tests mean, what medicines I am given, should I be careful….doctors in the government hospital should be available and give some time to me…otherwise these tests mean nothing… I have had diabetes and hypertension for the past 10-12 years, I also had a heart problem, I try to follow a healthy diet but I have stopped taking medicines, I cannot keep taking medicines forever unless someone explains to me why it is needed…..” (R9, male, 65 years, both diabetes and hypertension, not under control)

A few patients also shared that, sometimes to avoid the long waiting time at the health facility or due to limited time given by the doctor to them, they decided not to visit the health centers resulting in the intake of the same medicines over the years without consulting the doctor. Also, lack of information on the disease and the medicines prescribed often led to negligence in taking the medicines as advised.

“I am fed up with the waiting period in the hospitals and that is why I don’t go to the doctor…They don’t make any changes to the tablets. IfI the sugar level increases they just increase 5 points in the insulin, other than that they don't do anything. ……have been prescribed the same tablets for 3 years and I think they will give the same tablets for the next 10 years also (R12, female, 53 years, diabetic, not under control)

Interestingly, age also emerged as a barrier to practicing a healthy lifestyle. In-depth interviews with younger respondents revealed that they tend to think of ‘*here and now* rather than distant future consequences. Other than their carefree attitude, how their peer group might judge them was more important to them compared to their present health needs. Further, they hid their disease status from their family, friends and as a result received no support from them in disease management.

“I am young…this is the age to eat and drink… all this blood pressure and sugar are old people’s disease, I have to work and look after my family…I have not told about my health problems to anyone in family or friends…what people will say!!.” (R13, male, 41 years, both diabetes and hypertension, not under control)

“At this age only I am suffering from the disease but it’s okay !! I am not like others. I'm not scared of anything…I eat everything and do everything I want. I have not told about my diabetes condition to anyone in my family or friends…I have many things to do in life…I have to build a home and get both of my sons married” (R14, female, 46 years, widow, diabetic, not under control)

Further analysis from the narratives of the young people shows that the onset of chronic diseases like diabetes and hypertension and the recommended changes in lifestyle that come along with them often cause many young adults to feel “older” than their biological age. In-depth interviews show that often young people perceive it as shameful and the belief of their own identity and self-image as a “young” person is negatively altered. As a result, neither they like to adhere to any medication or lifestyle modifications nor seek any support from family or friends. Moreover, they try to hide it from their families and friends perceiving the age-related stigma of such diseases that may hamper their personal or working life.

The frontline health workers (FLW) and the counsellor played a pivotal role in the intervention. Their regular interaction and involvement with the patients brought many insights. The FLWs shared that often patients do not go for walks or exercise due to reasons such as inability to take time out of their daily routine, poor weather conditions or lack of motivation to go out alone. Also, some of the cultural food practices like ‘*gudde mamsa*’ where the whole community shares meat for eating were quite common. In certain rural communities in Karnataka, the practice of ‘*gudde mamsa*” is common. In this community practice, families do not buy meat just for themselves, rather they contribute to buying in bulk and each family gets 4–5 kg of meat (mostly mutton/red meat) for consumption. Sometimes, they also save money for this which they call ‘*mamsada cheeti*’ (a kind of chit fund for buying meat) and then they divide the meat through traditional ways among village communities. Such practices led to frequent and higher consumption of red meat than recommended. Moreover, among men smoking and drinking alcohol were also common in addition to this high consumption of meat.

## Enablers of better control of diabetes and hypertension

### Family support

A thorough study into each of the qualitative cases shows that those who had good control levels of blood pressure and/or sugar had also a strong support system with a cordial relationship between the family members. Strong family support not only motivated the patients to take better care of themselves but also provided emotional support to the patient in times of stressful events. Also, they ensured regular check-ups, proper, timely intake of medicines and help in making better lifestyle modifications such as preparing food compatible for diabetes and hypertension patients and motivating them to exercise. *R15* shared her experience of how the family support motivated and helped her adhere to the treatment regimens for a long time. But we also found that in most cases, family’s involvement remains very less mostly due to a lack of awareness about patients' health conditions.

“I am perfectly fine…I am doing everything as per the doctor’s advice. I go out for a walk, practice yoga, do meditation, eat a balanced diet and go for regular checkups and medication refills. A few years back I had some severe health issues (ARCUPPA), I was operated on for that, my husband and children have supported me throughout my critical times… there was a time when everyone thought I will die but with my family’s support, I am perfectly fine now….even now my husband makes herbal coffee every day, takes me for a walk, monitors my food and always motivates me…both my children are there to support me…my daughter calls me every day to remind me of the medicines” (R15, female, 63 years, hypertensive, controlled)

### Intrinsic motivation

Qualitative data showed that certain independent factors like the previous history of diabetes and hypertension in the family or witnessing the loss of someone due to these diseases pushed the patients to take better care of themselves. The trauma or grief of losing loved ones and witnessing negative life situations motivated patients to be cautious about their own lives.

“I used to work in the ICU as a support staff and every day I saw many patients suffering from different diseases and I don’t want to become like them and suffer…so I make sure that I do everything to control my sugar and blood pressure level” (R16, male, 63 years, both diabetes and hypertension, controlled)

“I have seen the deaths of my husband and mother-in-law. Nowadays I live alone here and if I feel sick also there is no one to take care of me… Hence I realised long back that I have to take care of myself so, I am taking tablets and doing yoga regularly” (R17, female, 83 years, hypertension, controlled)

Sometimes, life-threatening health events make the patient and their families cautious of their health and take better care of themselves. However, the above findings may vary, since it largely depends on an individual’s attitude towards life experiences.

“I can’t miss my tablets at any time. I have lost my vision in one eye due to my negligence, again I don’t want to take any chance and therefore I never miss the tablets” (R18, female, 50 years, both diabetes and hypertension, controlled)

## Discussion

This study was conducted as part of a larger study aiming to improve NCD continuum of care in an urban neighbourhood of Mysuru city, India. The study assessed multiple barriers, and enablers to control diabetes and hypertension using a mixed-methods study design. We found that longer duration, additional chronic conditions, tobacco consumption, and age play a major role in the control status of diabetes and for the hypertensive, higher BMI level majorly explains the variation. Further, the findings were elaborated by the qualitative assessment which shows the control status of the disease is affected by stress caused due to family or financial worries which are often ignored while designing the intervention programs. The qualitative findings indicate that stress, poor lifestyle and poor health-seeking behaviour interplay with other factors like age, occupation, economic constraints and absence of family support which led to poor control for diabetes and hypertension. Stress leading to poor self-care followed by poor diet, exercise and medication are the possible pathways for poor control.

The duration of the disease among diabetics had an important effect on the control level. It was observed that a majority of those with higher duration of disease could not achieve biological control status as compared to the ones with a relatively shorter duration. This may be explained through the lens of self-perception about the marginal hazard with time due to diabetes. People tend to get accustomed to the condition, therefore losing the rigor towards adherence to diabetes control [29–31]. Moreover, this could also be explained by progressive impairment of insulin metabolism over time because of beta-cell malfunction. This makes the response to diet alone or oral agents unlikely [32]. However, the curve for marginal hazard with time takes a reverse trend as the symptoms become serious. This can be observed in the findings from the qualitative study. People with higher disease duration reported attempts for adherence by adopting self-care practices but did not achieve the control status as the physical harm has taken a higher toll due to prolonged ignorance [33]. However, the findings do not suggest a similar explanation for hypertensive populations.

Being overweight puts a person at greater risk of hypertension which is further associated with an elevation in cardiac output [34]. The findings additionally show that overweight and obese people with hypertension had difficulty in maintaining control levels of blood pressure. However, increased BMI is also an indicator of poor lifestyle which we found to be quite profound in our study population.

Physical activity and consumption of fruits and vegetables did not play a significant role in the control of diabetes and hypertension. This is probably because the benefits of physical activity are mainly long-term rather than immediate [35]. However, our qualitative findings supported that a poor lifestyle is mainly caused by a lack of self-control or lack of motivation for healthy food habits, physical activity or any other lifestyle modifiers. Also, the nature of work and consumption of alcohol and/or smoking contributes to it. Moreover, the qualitative findings further indicate the need of the family in taking care of the patient and helping in disease management is enormous. It generally manifests through better food choices, eating patterns, timely medication and also emotional support [36]. We have also found higher consumption of tobacco and alcohol among patients with unstable familial relationships and those with poor social support. Despite being aware of the ill effects on health, patients continued to consume tobacco and alcohol, these findings emphasize that family support and continuous care provision are very important [30, 37–39].

Knowledge about the health benefits of fruit and vegetable consumption did not result in its increased intake, suggesting that knowledge alone may not be sufficient to trigger the behavioural change in the population [40]. Intake of food items was affected by choice, cultural practices, long-term habits, and economic status. Hence, patient-specific diet counselling after taking into account social and economic inequalities will be desirable.

Therefore, physical activity or a healthy diet alone might not be directly impacting the control level of diabetes and hypertension but a balanced interplay between these enablers towards a better lifestyle is crucial. Continuing support from family and health care providers is important to target patients’ psychological barriers, especially for adhering to physical activity regimens and healthy diet patterns [41].

Qualitative findings revealed that stress interferes with the ability to self-management of these two diseases. The absence of support from family members in disease management further deteriorated the situation. Therefore, there is a need for a greater understanding of the effects of stress on diabetes and hypertension; at the same time, it is important to assess and address patient needs for psychosocial support. A systematic review of family-based diabetes self-management intervention studies found that family involvement is a requisite for improvement in patients’ self-efficacy, perceived social support, disease knowledge, and self-care [36, 42–44]. As per recent evidence, integrative approaches such as yoga and meditation helps both in preventing [45] and achieving biological control of diabetes and hypertension [46] as well as in addressing stress and wellbeing [47]. These approaches are currently being piloted as part of the NCD continuum of care interventions in urban Mysuru. Health and Wellness Centres (HWCs) as part of the GOI’s Ayushmaan Bharat Program are able platforms to provide these integrative care solutions in addition to routine clinical care closer to the community and patient homes. As part of our intervention, we are incorporating them into the study area.

Lack of appropriate knowledge about the disease, risk factors and treatment adherence were more frequent among those with poor control levels. The findings are in accordance with similar previous research evidence[48–51]. Based on the findings, it can be said that adults living with diabetes and hypertension need proper knowledge of these risk factors to achieve better control and prevent complexity.

Assessment of both quantitative and qualitative findings indicates that medication only does not always ensure effective control of diabetes and hypertension [52]. The patients and clinicians should engage in the shared decision-making process to determine individualisation of treatment targets, with the acknowledgment that the benefits and risks of intensive HbA1c/SBP DBP targets are uncertain and may vary across patients [53, 54].

The current study explicitly flagged the role of individualistic factors in determining the control status of diabetes and hypertension. Measures such as HbA1c were used as an indicator of good and poor control of diabetes and provided an average blood glucose level during the last three months [55] Despite being considered the gold standard for assessing control levels of blood sugar among diabetics, the HbA1c test is not available in almost all government hospitals, especially in the urban PHCs. Besides, stress is not palpable; it requires a trained workforce to understand and be able to communicate with the patients to motivate them in finding ways to cope with the disease and stay healthy [56, 57]. UPHCs in India currently has an acute shortage of frontline health workers such as female community health activists/ Accredited Social Health Activists (ASHA) and the government faces challenges in the recruitment and retention of such staff [6]. Efforts such as patient support groups, health and wellness centres, and facility-based psychosocial counselling can go long way in complementing current efforts of NCD control at the population level. Findings from this study will help program officers to develop patient-centric treatment and wellness plans.

## Limitations and strengths of the study

This study holds some limitations. For the qualitative study, the limited number of UPHCs and associated sample size of the populations are a limitation and extrapolation of the findings to larger geography should be done carefully. Being a cross-sectional study design, it is difficult to draw a cause-and-effect relationship between factors assessed and control levels.

At the same time, this study was undertaken in the context of a program intervention to strengthen the NCD care continuum at PHC level. This has implications and lessons for how PHC level NCD program can be strengthened. Although samples were collected at the patient level, the information from the provider/caregiver's perspective brought additional value in terms of a comprehensive understanding of NCD management.

The study focuses on a wide range of determinants, socio-demographic, behavioural and clinical and hence provides crucial information to the NCD program managers and policymakers to explore how synergies between health and other development sectors such as education, communication, food, justice and security, environment, social welfare etc. can be realized. Such multisectoral action is essential for the formation of conducive environments to support healthy behaviours.

## Conclusion

This study adds to the limited information on possible factors and determinants associated with poor control of diabetes and hypertension in India, and the need to strengthen patient-specific care and family or community engagement approach across NCD continuum of care. This study is a follow-up to a population-based assessment in 2017–18 to design interventions for the prevention and management of diabetes and hypertension at an urban primary health centre [15]. The study assessed factors associated with blood sugar and blood pressure control within particular geography; this can help the government to have cost-effective and pragmatic solutions to address the growing burden of diabetes and hypertension. With a better understanding of factors associated with disease control, individualistic focused patient outreach plans, customised communication strategies, need-based care delivery plans and specific competency-based capacity building models for health care workers are to be developed. The study also reinforces the need to achieve convergence between health and other health-related sectors for comprehensive management of NCDs at the primary care level.

## Data Availability

The datasets used and/or analysed during the current study are available from the corresponding author on reasonable request.

## Abbreviations

PHC: Primary Health Care Settings
NCD: Non-Communicable Disease
HbA1c: Glycated Haemoglobin A1c
BP: Blood Pressure
IDI: In-Depth Interviews
BMI: Body Mass Index
MoHFW: Ministry of Health and Family Welfare
GOI: Government of India
NDCDCS: National Programme for Prevention and Control of Cancer, Diabetes, Cardiovascular Diseases and Stroke
WHO: World Health Organization
KHPT: Karnataka Health Promotion Trust
UPHC: Urban Primary Health Care Centre
CI: Confidence Interval
RBS: Random Blood Sugar
SBP: Systolic Blood Pressure
DBP: Diastolic Blood Pressure
ACR: Albumin to Creatinine Ratio Test
ECG: Electrocardiogram
FLW: Frontline Health Workers
ASHA: Accredited Social Health Activist
